# Survival after surgery among patients with cholangiocarcinoma in Northeast Thailand according to anatomical and morphological classification

**DOI:** 10.1186/s12885-021-08247-z

**Published:** 2021-05-03

**Authors:** Chaiwat Tawarungruang, Narong Khuntikeo, Nittaya Chamadol, Vallop Laopaiboon, Jaruwan Thuanman, Kavin Thinkhamrop, Matthew Kelly, Bandit Thinkhamrop

**Affiliations:** 1 Epidemiology and Biostatistics Program, Faculty of Public Health, Khon Kaen University, Khon Kaen, Thailand; 2 Cholangiocarcinoma Screening and Care Program (CASCAP), Faculty of Medicine, Khon Kaen University, Khon Kaen, Thailand; 3 Data Management and Statistical Analysis Center (DAMASAC), Faculty of Public Health, Khon Kaen University, Khon Kaen, Thailand; 4 Cholangiocarcinoma Research Institute (CARI), Khon Kaen, Thailand; 5 Department of Surgery, Faculty of Medicine, Khon Kaen University, Khon Kaen, Thailand; 6 Department of Radiology, Faculty of Medicine, Khon Kaen University, Khon Kaen, Thailand; 7 Health and Epidemiology Geoinformatics Research (HEGER), Faculty of Public Health, Khon Kaen University, Khon Kaen, Thailand; 8 Department of Global Health, Research School of Population Health, Australian National University, Canberra, Australia; 9 Epidemiology and Biostatistics Section, Faculty of Public Health, Khon Kaen University, Khon Kaen, 40002 Thailand

**Keywords:** Cholangiocarcinoma, Anatomical, Morphological, Classification, Survival, CASCAP

## Abstract

**Background:**

Cholangiocarcinoma (CCA) has been categorized based on tumor location as intrahepatic (ICCA), perihilar (PCCA) or distal (DCCA), and based on the morphology of the tumor of the bile duct as mass forming (MF), periductal infiltrating (PI) or intraductal (ID). To date, there is limited evidence available regarding the survival of CCA among these different anatomical and morphological classifications. This study aimed to evaluate the survival rate and median survival time after curative surgery among CCA patients according to their anatomical and morphological classifications, and to determine the association between these classifications and survival.

**Methods:**

This study included CCA patients who underwent curative surgery from the Cholangiocarcinoma Screening and Care Program (CASCAP), Northeast Thailand. The anatomical and morphological classifications were based on pathological findings after surgery. Survival rates of CCA and median survival time since the date of CCA surgery and 95% confidence intervals (CI) were calculated. Multiple cox regression was performed to evaluate factors associated with survival which were quantified by hazard ratios (HR) and their 95% CIs.

**Results:**

Of the 746 CCA patients, 514 had died at the completion of the study which constituted 15,643.6 person-months of data recordings. The incidence rate was 3.3 per 100 patients per month (95% CI: 3.0–3.6), with median survival time of 17.8 months (95% CI: 15.4–20.2), and 5-year survival rate of 24.6% (95% CI: 20.7–28.6). The longest median survival time was 21.8 months (95% CI: 16.3–27.3) while the highest 5-year survival rate of 34.8% (95% CI: 23.8–46.0) occurred in the DCCA group. A combination of anatomical and morphological classifications, PCCA+ID, was associated with the longest median survival time of 40.5 months (95% CI: 17.9–63.0) and the highest 5-year survival rate of 42.6% (95% CI: 25.4–58.9). The ICCA+MF combination was associated with survival (adjusted HR: 1.45; 95% CI: 1.01–2.09; *P* = 0.013) compared to ICCA+ID patients.

**Conclusions:**

Among patients receiving surgical treatment, those with PCCA+ID had the highest 5-year survival rate, which was higher than in groups classified by only anatomical characteristics. Additionally, the patients with ICCA+MF tended to have unfavorable surgical outcomes. Showed the highest survival association. Therefore, further investigations into CCA imaging should focus on patients with a combination of anatomical and morphological classifications.

**Supplementary Information:**

The online version contains supplementary material available at 10.1186/s12885-021-08247-z.

## Background

Cholangiocarcinoma (CCA) is the most common primary malignancy of the biliary tract [1]. CCA is relatively rare worldwide, but very high incidence rates have been reported in East and Southeast Asia, this is especially the case in Thailand [2], which has an estimated 6 million people infected with the liver fluke, *Opisthorchis viverrini* which is the major risk for developing CCA in this area [3]. In Thailand, CCA incidence has been recorded as being up to 87.7 per 100,000 in males and 36.3 per 100,000 in females [4]. It is the most common primary liver cancer in the northeast of Thailand where it has its highest incidence worldwide [5, 6], and it is also one of the major causes of death. In comparison, the 2010–2012 age-standardized incidence rate of CCA worldwide was 53.4 per 100,000 population in males and 18.5 in females [7].

To address these on-going, serious health problems, a series of projects have been implemented to improve screening for CCA in high-risk populations. The first project was a screening program performed in Ban Luang District, Nan Province, Northern Thailand which found that the detection of early-stage CCA by ultrasonography (US) resulted in an improved clinical outcome [8]. Another program that was introduced was the Cholangiocarcinoma Screening and Care Program (CASCAP) which continues to screen high risk populations in high incidence areas in order to detect patients in with early stage CCA. A recent study from CASCAP showed that US screening was an effective tool for detecting early-stage CCA [9].

CCA has been classified based on the anatomic location of the bile duct tumor which is categorized as intrahepatic CCA (ICCA), perihilar CCA (PCCA) or distal CCA (DCCA) [10]. ICCA is the second-most common primary liver cancer; its incidence having increased by 22% between 1979 and 2004 [1]. The incidence of extrahepatic CCA increased from 2001 to 2007, coinciding with the 2001 implementation of a new version of the International Classification of Diseases for Oncology. Reanalysis of previous registries indicated that PCCA is often misclassified as ICCA, thus, the increase in ICCA reported by many registries could result from an increase in PCCA [11]. Previous studies have reported the survival of patients with ICCA after surgery in Thailand where the cumulative 1, 3, and 5-year survival rates were 52.1, 21.7, and 11.2%, respectively. The median duration of survival after resection was 12.4 months [12]. In comparison, survival of patients after resection of PCCA in the United States and Western Europe, had a median overall survival of 19 months and an estimated 1, 3 and 5-year survival of 69, 27 and 13%, respectively [13]. Conditional survival for patients with unresectable PCCA in the Netherlands between 2002 and 2012 had overall survival of 42% at 1-year and 6% at 3-year [14]. The survival of resected and unresected DCCA patients in the Netherlands between 2009 and 2016, had a median overall survival of 10.4 months across all stages; 21.9 months for resected, 6.7 months for unresected nonmetastatic, and 3.6 months for metastatic DCCA with the *P* was < 0.001 [15]. In 2019, there was a study performed in 11,710 CCA patients from Surveillance, Epidemiology, and End Results Cancer Registries (SEER) compared survival for ICCA, PCCA, and DCCA and found that 5-year overall survival was highest in ICCA (16.7%) followed by PCCA (16.4%) and DCCA (5.7%) [16].

In addition, CCA can be classified into mass-forming (MF), periductal-infiltrating (PI) and intraductal (ID) [17]. Previous studies have found a relationship between tumor location and tumor morphology in CCA patients. One study based on the survival analyses of four groups of ICCA according to tumor morphology: ID, PI, MF and PI plus MF, found that overall survival at 1, 5, and 10 years was 50, 36, and 36%, respectively. Moreover, this study also found a poor outcome in the case of MF with PI with the stage of CCA of IV-A [18].

Importantly, the very high mortality of CCA patients was directly related to difficulties in diagnosing the disease at an early stage when surgical cure is possible [19]. CCA is very difficult to detect and diagnose, and has a very poor prognosis with a 5-year survival rate of less than 5% [20]. Most CCA patients come to a physician at a late stage of the disease, as the tumors are clinically silent in the early stages [21, 22]. However, there is limited evidence available regarding the survival rate of patients with different anatomical and morphological characteristics of CCA from the median time/date of surgery until patient death. Furthermore, there is limited evidence regarding those factors that are associated with the survival of CCA patients in different anatomical and morphological classifications.

Therefore, our study aimed to evaluate the survival rate and median survival time after surgery among patients with CCA according to anatomical and morphological classification and to determine whether there was an association of anatomical and morphological classification with survival after surgery in the northeast of Thailand.

## Methods

### Design overview

**Fig. 1:**
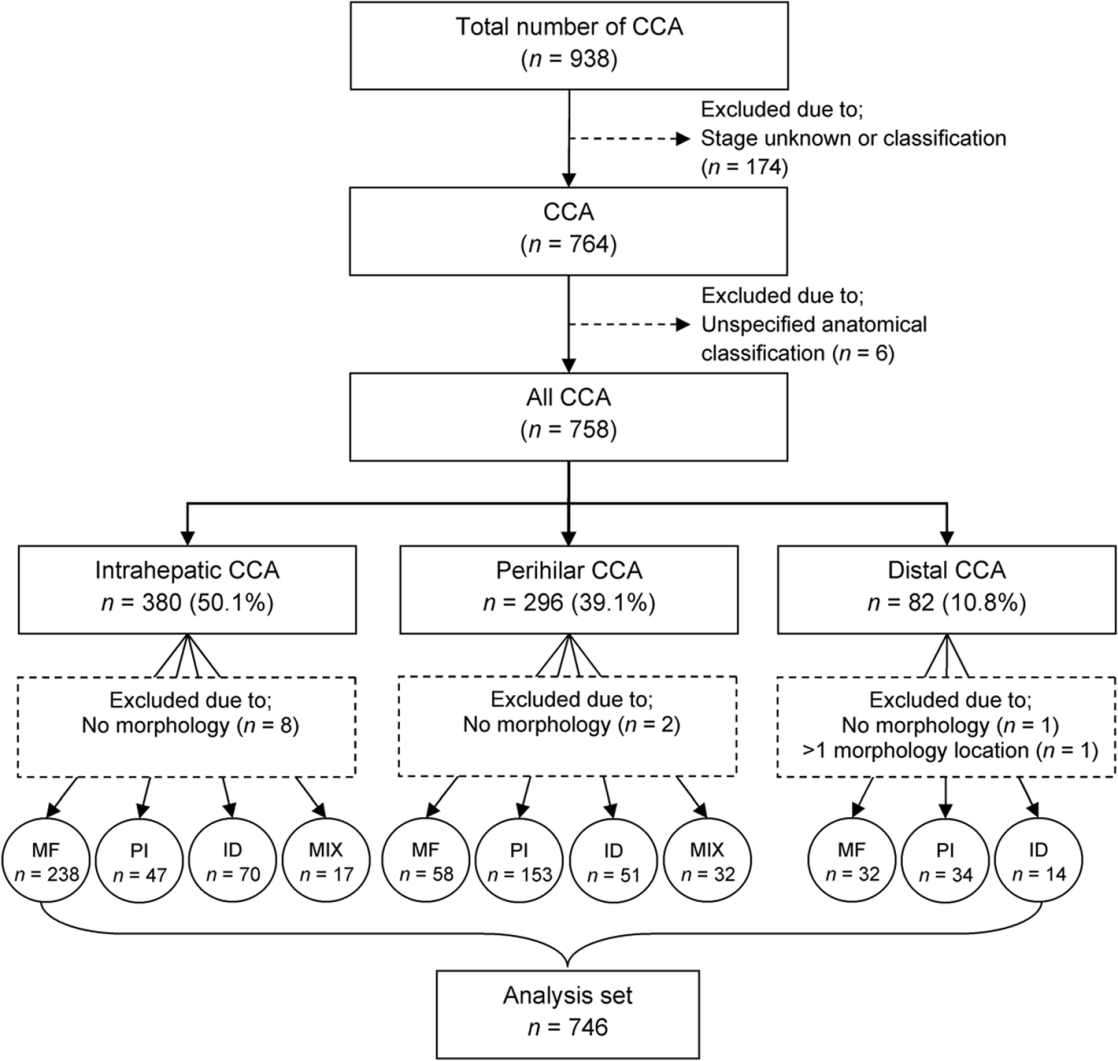
Sample selection process. CCA: Cholangiocarcinoma; ID: Intraductal; MF: Mass-forming; MIX: Morphological > 1; PI: Periductal-infiltrating

This study uses a prospective design, monitoring CCA patients who were diagnosed by biopsy and subsequently underwent resection for CCA until their final stage of life. The data from the Cholangiocarcinoma Screening and Care Program (CASCAP), northeast Thailand, were analyzed to evaluate the survival rate, median survival time, and factors that are associated with the survival of patients with different CCA anatomical and morphological classifications. All participants were from both screening and walk-in groups enrolled in the CASCAP database between 2013 and 2019. The screening group were participants who underwent routine ultrasonography screening for CCA but showed no symptoms that could be related to CCA. The walk-in group were participants presenting at the hospital with clinical symptoms suggestive of CCA as a possible cause. Both screening and walk-in groups were diagnosed for suspected CCA. Patients with the pre-operative findings that indicated a potential to be cured by surgical treatment such as having no distance metastasis or appearing to be resectable were provided a curative-intent operation. Patients in this category are definitely early stage (stage 0, I, and II) and are included in the study. We did not include patients with late stage (stages III and IV). The actual staging in our study was based on the findings after completion of the operation where the clinical and/or pathological characteristics can be correctly determined. Data on histological findings were based on the standard protocol of the tertiary hospital at Khon Kaen University, Thailand. The CCA patients with an unspecified anatomical classification and/or unknown stage of CCA, and/or the date of CCA diagnosis being incomplete were excluded from further analyses. This resulted in a total of 746 CCA cases which were included in the study following the sampling technique shown in Fig. 1.

### Primary outcomes and study factors

The primary outcome for this study was the elapsed time (number of months) from the date of surgery for CCA (begin date) to the date of death or last follow-up (end date). The median follow-up period was 17.2 months with a total of five-year follow-up period. The factors of interest were the anatomical and morphological classifications which were categorized into 11 groups, namely, ICCA+MF (ICM), ICCA+PI (ICP), ICCA+ID (ICI), ICCA+Mix morphology (ICMIX), PCCA+MF (PCM), PCCA+PI (PCP), PCCA+ID (PCI), PCCA+Mix morphology (PCMIX), DCCA+MF (DCM), DCCA+PI (DCP), and DCCA+ID (DCI). The other factors included gender, age at enrolment, education levels, occupation, history of *O. viverrini* infection, history of praziquantel treatment, and stage of CCA. The CCA staging defined as early and late stage which early stage refers to stage 0, I, II, and late stage refers to stage III and IV according to the 7th edition AJCC staging [23, 24].

### Statistical analyses

The baseline characteristics of CCA patients were described using frequency and percentage for categorical data stratified by the anatomical and morphological classification of CCA. The mean, standard deviation (SD), median, and minimum and maximum range for continuous data were recorded.

The CCA incidence rate per 100 person-months since surgery with CCA and its 95% confidence interval (CI) were calculated based on Poisson distribution assumptions. The survival rate and median survival time since the date of surgery with CCA until death by CCA and its 95% CI were estimated using Kaplan-Meier methods. The association between the anatomical and morphological classification and CCA survival adjusted for other factors was quantified by adjusted hazard ratio (HR) and its 95% CI using multiple cox regression analysis. A *P*-value of less than 0.05 was considered to be significant. All analyses were performed using STATA version 15 (StataCorp, College Station, TX).

## Results

A total of 746 CCA patients were included in the analysis with a mean age of 61.1 ± 8.5 years, ranging from 34 to 84 years, more than half (54.6%) were > 60 years old (Table 1). Around two-thirds were male and were in late stage of CCA (63.9 and 65.3%, respectively). According to the combination of anatomical and morphological classifications, the largest group, 31.9% (238/746), of patients were in the ICM group followed by 20.5% (153/746) of patients in the PCP group. The DCI group contained the lowest percentage of patients at 1.9% (14/746).

**Table 1:** Demographic characteristics of cholangiocarcinoma patients according to the anatomical and morphological classifications

Characteristics	Overall		Intrahepatic				Perihilar				Distal		
			MF	PI	ID	Mix	MF	PI	ID	Mix	MF	PI	ID
	***n*** = 746	%	***n*** = 238	***n*** = 47	***n*** = 70	***n*** = 17	***n*** = 58	***n*** = 153	***n*** = 51	***n*** = 32	n = 32	***n*** = 34	***n*** = 14
Gender													
Female	269	36.1	95	18	24	8	15	39	17	13	14	18	8
Male	477	63.9	143	29	46	9	43	114	34	19	18	16	6
Age													
< 50	68	9.1	26	2	3	1	3	21	5	1	2	4	0
50 to 60	271	36.3	93	16	19	7	20	52	21	13	15	11	4
> 60	407	54.6	119	29	48	9	35	80	25	18	15	19	10
Mean (SD)	61.1 (8.5)		60 (8.4)	63.4 (8.5)	63.4 (8.4)	61 (7.4)	62.3 (7.6)	60.1 (9.2)	61.3 (8.4)	61.4 (7.4)	60.8 (8.9)	62.1 (9.1)	64 (5.6)
Range	34–84		34–84	46–81	42–82	47–72	43–80	36–82	41–76	44–77	41–78	40–77	55–74
Education													
Primary and lower	615	82.4	199	39	61	14	43	127	42	24	26	29	11
Secondary	67	9.0	19	3	6	1	7	17	4	4	4	2	0
Certificate and higher	64	8.6	20	5	3	2	8	9	5	4	2	3	3
Occupation													
Unemployed	33	4.4	8	3	3	0	3	7	2	2	1	3	1
Farmer	502	67.3	165	32	50	12	37	102	32	23	21	18	10
Others	211	28.3	65	12	17	5	18	44	17	7	10	13	3
Smoking history													
No	360	48.3	126	24	36	9	24	56	22	17	16	21	9
Yes	386	51.7	112	23	34	8	34	97	29	15	16	13	5
Drinking history													
No	221	29.6	79	15	23	6	14	33	14	9	9	16	3
Yes	525	70.4	159	32	47	11	44	120	37	23	23	18	11
*Opisthorchis viverrini*													
No	560	75.1	171	38	50	13	40	119	37	24	28	30	10
Yes	186	24.9	67	9	20	4	18	34	14	8	4	4	4
Praziquantel treatment													
No	415	55.6	131	28	39	9	25	81	27	18	23	24	10
Yes	331	44.4	107	19	31	8	33	72	24	14	9	10	4
Stage of CCA													
Early stage	259	34.7	80	23	44	3	5	44	30	3	8	11	8
Late stage	487	65.3	158	24	26	14	53	109	21	29	24	23	6

### Survival analysis

**Fig. 2:**
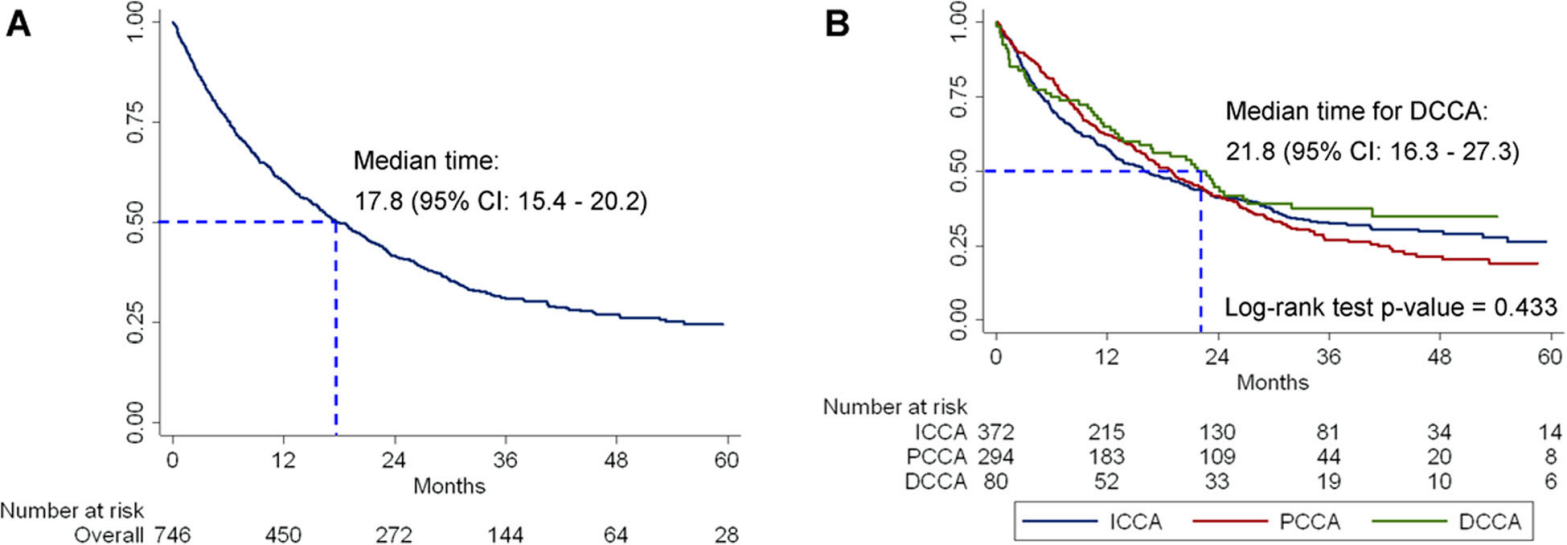
Kaplan-Meier survival estimates of cholangiocarcinoma. **a** Overall survival. **b** Survival according to the anatomical classification. ICCA: intrahepatic cholangiocarcinoma; PCCA: perihilar cholangiocarcinoma; DCCA: distal cholangiocarcinoma

**Fig. 3:**
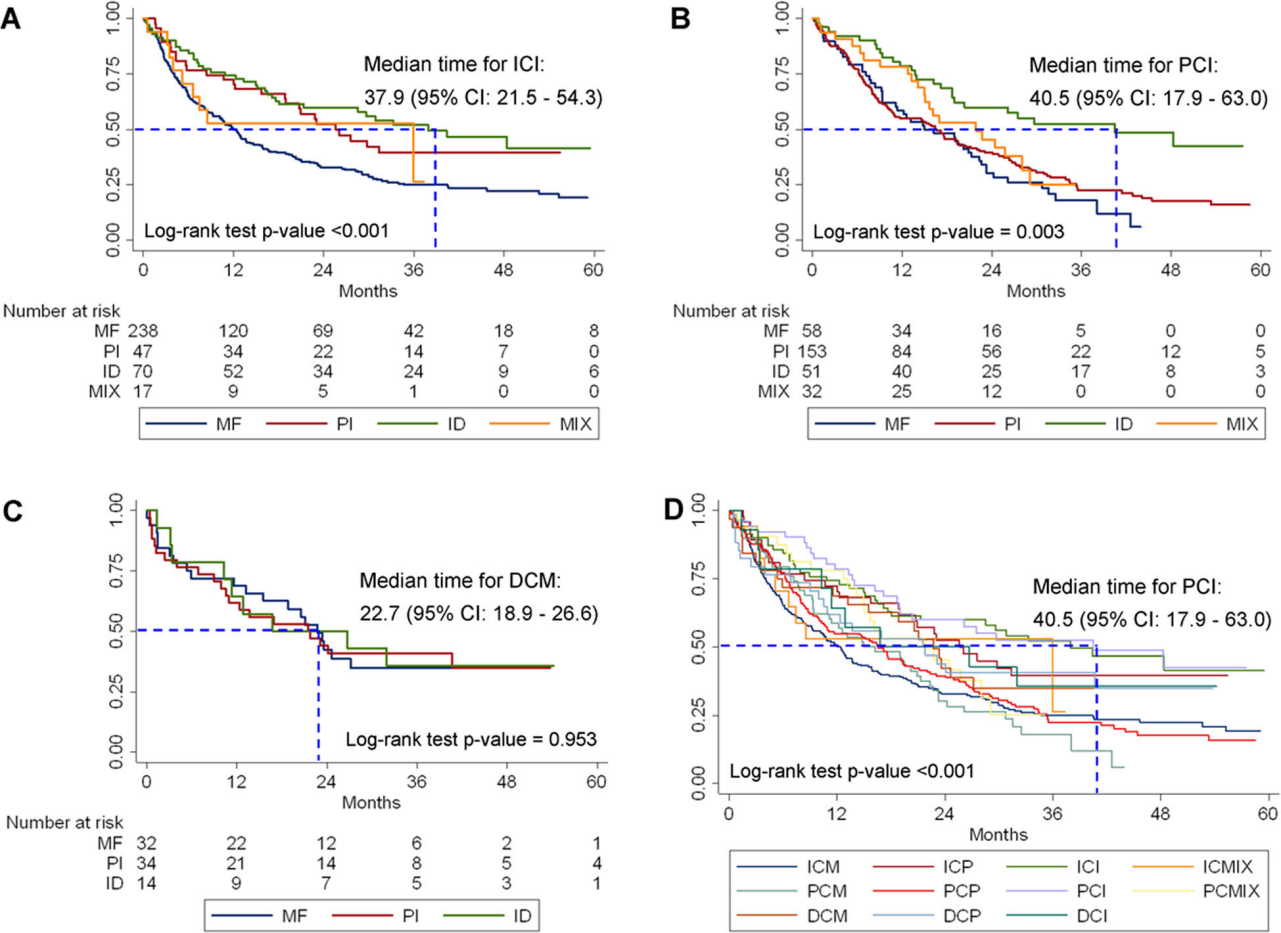
Kaplan-Meier survival estimates of cholangiocarcinoma according to the morphological classification. **a** Intrahepatic cholangiocarcinoma. **b** Perihilar cholangiocarcinoma. **c** Distal cholangiocarcinoma. **d** All combinations of anatomical and morphological classifications. MF: Mass-forming; PI: Periductal-infiltrating; ID: Intraductal; MIX: More than one morphological; ICM: Intrahepatic+Mass-forming; ICP: Intrahepatic+Periductal-infiltrating; ICI: Intrahepatic+Intraductal; ICMIX: Intrahepatic+Mix morphological; PCM: Perihilar+Mass-forming; PCP: Perihilar+Periductal-infiltrating; PCI: Perihilar+Intraductal; PCMIX: Perihilar+Mix morphological; DCM: Distal+Mass-forming; DCP: Distal+Periductal-infiltrating; DCI: Distal+Intraductal

Of a total of 746 CCA patients, constituting of 15,643.6 person-months of observations, 514 patients died after surgery. The overall incidence rate was 3.3 per 100 patients per month (95% CI: 3.0–3.6). According to the combination of anatomical and morphological classifications, the highest incidence rate was 4.7 per 100 patients per month (95% CI: 3.6–6.3) found in the PCM group, followed by patients with ICM with 4.1 per 100 patients per month (95% CI: 3.6–4.8), and for the PCP group 3.8 per 100 patients per month (95% CI: 3.2–4.6) (Table 2). The overall median survival time after surgery was 17.8 months (95% CI: 15.4–20.2). According to the anatomical classification, the median survival was statistically not significantly different with a log-rank test *P* = 0.433 (Fig. 2). According to the combination of anatomical and morphological classifications, the median survival time for CCA patients after surgery was highly statistically significantly different across groups with a log-rank test *P* < 0.001. The patients with PCI had the longest median survival time (40.5 months; 95% CI: 17.9–63.0), whereas the shortest was found in patients with ICM (12.4 months; 95% CI: 10.2–14.6) (Fig. 3).

**Table 2:** Incidence rate and median survival time of cholangiocarcinoma patients according to the anatomical classification and a combination of anatomical and morphological classifications

Cholangiocarcinoma	Number	Person-months	IR per 100	Median time	95% CI
Overall	746	15,644	3.3	17.8	15.4–20.2
Anatomical classifications					
ICCA	372	7714	3.3	16.2	12.3–20.2
PCCA	294	6065	3.5	19.0	15.8–22.2
DCCA	80	1865	2.7	21.8	16.3–27.3
Combination of anatomical and morphological classification					
ICM	238	4373	4.1	12.4	10.2–14.6
ICP	47	1161	2.3	25.6	18.9–32.4
ICI	70	1885	1.9	37.9	21.5–54.3
ICMIX	17	294	3.1	36.0	02.0–69.9
PCM	58	990	4.7	16.2	08.2–24.3
PCP	153	3074	3.8	16.5	11.0–22.0
PCI	51	1397	1.9	40.5	17.9–63.0
PCMIX	32	605	3.5	21.9	11.8–31.9
DCM	32	675	3.0	22.7	18.9–26.6
DCP	34	821	2.6	21.5	08.2–34.8
DCI	14	369	2.4	16.8	08.6–42.2

*IR* Incidence rate, *CI* Confidence interval

**Fig. 4:**
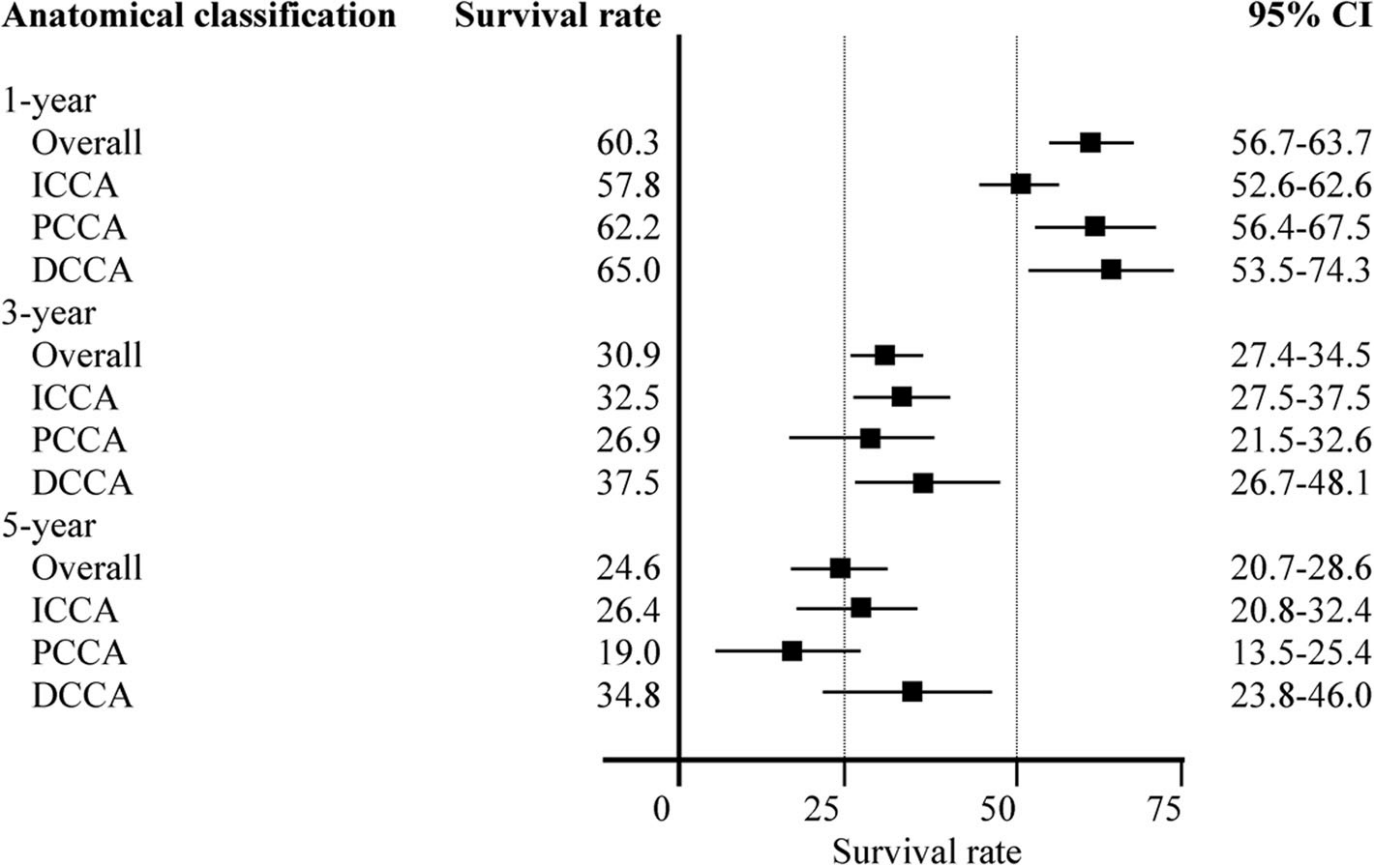
One-year, three-year, and five-year survival rates of CCA according to the anatomical classification. CCA: Cholangiocarcinoma; CI: Confidence interval; ICCA: Intrahepatic CCA; PCCA: Perihilar CCA; DCCA: Distal CCA

**Fig. 5:**
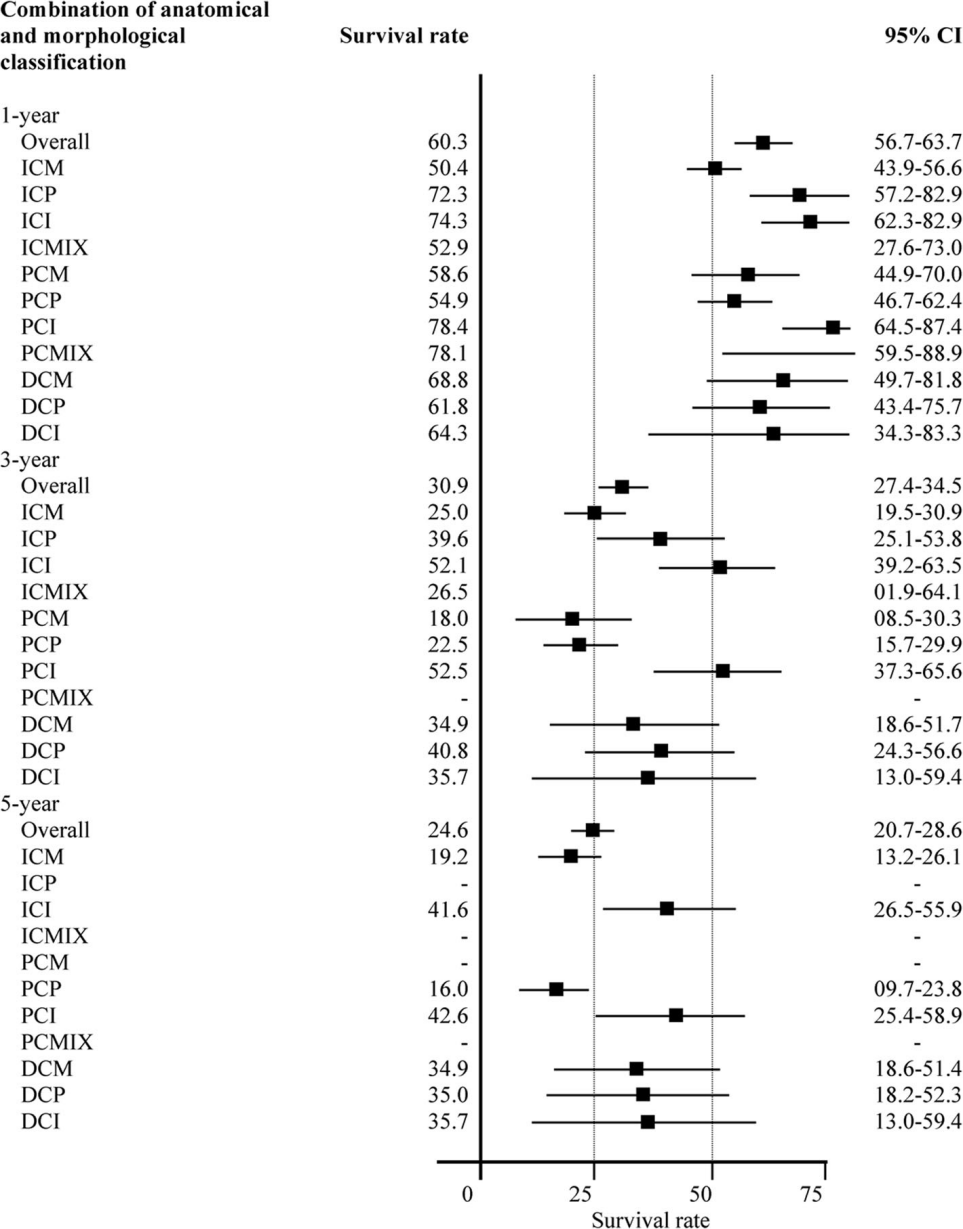
One-year, three-year, and five-year survival rates for cholangiocarcinoma according to the anatomical and morphological classifications. CI: Confidence interval; ICM: Intrahepatic+Mass-forming; ICP: Intrahepatic+Periductal-infiltrating; ICI: Intrahepatic+Intraductal; ICMIX: Intrahepatic+Mix morphological; PCM: Perihilar+Mass-forming; PCP: Perihilar+Periductal-infiltrating; PCI: Perihilar+Intraductal; PCMIX: Perihilar+Mix morphological; DCM: Distal+Mass-forming; DCP: Distal+Periductal-infiltrating; DCI: Distal+Intraductal

The overall survival rate was 60.3% (95% CI: 56.7–63.7) at 1 year, 30.9% (95% CI: 27.4–34.5) at 3 years, and 24.6% (95% CI: 20.7–28.6) at 5 years. According to the anatomical classification, the highest survival rate at 5 years occurred in DCCA patients (34.8%; 95% CI: 23.8–46.0) (Fig. 4). According to the anatomical and morphological classification of CCA, the highest survival rate at 1, 3, and 5 years occurred in PCI patients namely, 78.4% (95% CI: 64.5–87.4), 52.5% (95% CI: 37.3–65.6), and 42.6% (95% CI: 25.4–58.9), respectively, whereas the lowest rate at 1, 3, and 5 years occurred in 50.4% (95% CI: 43.9–56.6) for the ICM group, 18% (95% CI: 8.5–30.3) for the PCM group, and 16% (95% CI: 9.7–23.8) for the PCP group (Fig. 5).

### Bivariate and multivariable survival analysis

Bivariate results following cox regression analyses, according to the anatomical classification, showed no significant associations for patients in the PCCA and DCCA groups. After controlling for the effect of CCA staging, gender, age, history of *O. viverrini* infection and other factors, multivariable analyses showed a significant association with survival in patients with DCCA (HR: 0.67; 95% CI: 0.49–0.91) compared to the ICCA group (Table 3). According to the combination of anatomical and morphological classifications for CCA, bivariate results showed significant associations for patients in the ICM, PCM, and PCP groups. After controlling for the effect of CCA staging, gender, age, history of *O. viverrini* infection and other factors, multivariable analyses showed a significant association with survival in patients with ICM (HR: 1.45; 95% CI: 1.01–2.09) compared to the ICI group (Table 4).

**Table 3:** Multivariable analyses for the anatomical classification with survival in CCA patients using multiple cox regression

Anatomical classification	Person-months	IR per 100	Crude HR	Adjusted **HR	95% CI	***P***-value
ICCA	7714	3.3	Ref.	Ref.		0.024
PCCA	6065	3.5	1.03	0.85	0.70–1.03	
DCCA	1865	2.7	0.84	*0.67	0.49–0.91	

HR: hazard ratio; Ref.: reference group

* *P* < 0.05

** Hazard ratio of anatomical classification adjusted for effects of stage of CCA, gender, age, *O. viverrini* infection, education levels, occupation, smoking cigarettes, alcohol drinking, and praziquantel treatment

**Table 4:** Multivariable analyses for the combination of anatomical and morphological classification with survival in CCA patients using multiple cox regression

Combination of anatomical and morphological classification	Person-months	IR per 100	Crude HR	Adjusted **HR	95% CI	***P***-value
ICI	1885	1.9	Ref.	Ref.		0.013
ICM	4373	4.1	*2.03	*1.45	1.01–2.09	
ICP	1161	2.3	1.18	0.96	0.58–1.59	
ICMIX	294	3.1	1.39	0.84	0.40–1.77	
PCM	990	4.7	*2.13	1.12	0.72–1.75	
PCP	3074	3.8	*1.88	1.23	0.84–1.81	
PCI	1397	1.9	0.96	0.84	0.51–1.40	
PCMIX	605	3.5	1.55	0.80	0.46–1.39	
DCM	675	3.0	1.44	0.74	0.42–1.29	
DCP	821	2.6	1.35	0.91	0.52–1.58	
DCI	369	2.4	1.30	1.05	0.50–2.21	

*CI* Confidence interval, *HR* hazard ratio, *Ref*. reference group

* *P* < 0.05

** Hazard ratio of anatomical classification adjusted for effects of stage of CCA, gender, age, *O. viverrini* infection, education levels, occupation, smoking cigarettes, alcohol drinking, and praziquantel treatment

## Discussion

Cholangiocarcinoma is the most common primary malignancy of the biliary tract [1], with very high incidence rates reported in East and Southeast Asia, especially in Thailand [2]. The incidence of CCA has been reported to be as high as 87.7 per 100,000 in males and 36.3 per 100,000 in females [4].

Our study analyzed the pathological characteristics of CCA depending on its anatomical location (ICCA, PCCA, and DCCA) and morphological characteristics (MF, PI, ID) in order to evaluate the survival rate and median survival time after surgery, as well as to determine the association between these classifications and survival for a total of 746 CCA patients from northeast Thailand. Our results showed that most CCA patients were male (63.9%), almost twice as many as females, while the at risk population that came for CCA screening were predominately females. More than half of the 746 patients (54.6%) were elderly (aged greater than 60 years old), which is consistent with previous studies [25]. In addition, approximately two-thirds of CCA patients were found to be in late stages of the disease (65.3%) presenting with symptoms indicating CCA. This may be because the CCA screening program (CASCAP) is not comprehensive and that patients are asymptomatic, consequently they present at medical facilities when the disease has progressed and symptoms become apparent. The combination of anatomical and morphological classifications showed that the percentage of patients in the ICM group was highest (31.9%), whereas the lowest percentage was for patients in DCI group with about 2%.

The survival analyses showed that the overall incidence rate was 39.6 per 100 patients per year. The highest rate was found in the PCM group followed by the ICM and PCP groups (56.4, 49.2, and 45.6 per 100 patients per year, respectively). Likewise, compared to the incidence rate according to the anatomical classification alone, our study also found the highest rate to be in the PCCA group (42 per 100 patients per year); however, the rate was lower when classified by both anatomical and morphological classifications. In our series, half of all patients, 49.9% (372/746), were ICCA. Among these, three quarters, 64.0% (238/372) were mass forming (ICM) and only 12.6% (47/372) were periductal infiltrating (ICP). Such distribution was consistent with previous studies [26]. The incidence rate of ICM was approximately double that of the ICP, i.e., 4.1 versus 2.3 per 100 person-months. This was contrast to what was found in a study by Uno and colleagues [27]. Although this was based on only 16 patients who had ICP, this discrepancy addressed issues for improving surgical outcome among this group of patients.

The overall median survival times was about 18 months, the longest median survival time was 40.5 months for PCI patients, which was different when only classified anatomically when the longest median survival time was in DCCA patients (21.8 months). The shortest median survival time was found in patients with ICM (12.4 months) which was consistent with ICCA patients without morphological classification (16.2 months). The incidence rate and median survival time according to anatomical classification, and a combination of anatomical and morphological classifications separated by stage of CCA, are detailed in Additional file 1 (Table S1, Figs. S1, S2, S3, and S4). The overall survival rate was 60% at 1-year, 31% at 3-years, and 25% at 5-years. The highest survival rate was found in the patients with PCI 78.4, 52.5, and 42.6% at 1-year, 3-years, and 5-years, respectively. These rates were different when classified only anatomically, when the highest survival rate in patients with DCCA was 65% at 1 year, 37.5% at 3 years, and 34.8% at 5 years, respectively. Our finding is contrast with what was found in a study by Hang and colleagues in 2019 which showing that the highest 5-year overall survival was found in ICCA (16.7%) followed by PCCA (16.4%) and DCCA (5.7%) [16]. The large difference of the 5-year survival rate among DCCA patients between this and our study, 5.7% versus 34.8%, could caused by various factors such as cause of the disease, cancer staging at time of surgery, surgical resectability, susceptibility to lymph node dissection, etc. There is little evidences for this paper and ours. Obviously, the proportion of patient with late stage CCA in this and our study were 59.8% versus 65.3% which indicated that our study had a higher favorable surgical outcome even having a lower proportion of patients in the late. But what really plays roles here remained unclear to us. Indeed, there are limited consensus evidences regarding the factors that strongly affect surgical outcome among CCA patients. The lowest survival rate at 5-years was found in PCP patients (16%), which is lower than for PCCA patients (19%) who were classified only by anatomical characteristics. The survival rate according to the anatomical classification, and a combination of anatomical and morphological classifications separated by the stage of CCA, found that patients with early stage disease have higher survival rates than those with late stage disease (see Additional file 1 Figs. S5, S6, and S7). This is consistent with a 5-year population-based study conducted in north Thailand in 2011 which found that CCA patients with stage 0 had a 100% 3-year survival rate [8].

The association between anatomical classification and survival using cox regression, bivariate analyses found no significant associations for all groups. After controlling for the effect of other factors (stage of CCA, gender, age at enrollment, history of *O. viverrini* infection, education levels, occupation, cigarettes smoking history, alcohol consumption history, and praziquantel treatment) that have been previously reported as associated with CCA [25, 28, 29], significant associations were found in DCCA patients. According to a combination of anatomical and morphological classifications, the association of survival using simple cox regression analysis found significant associations for the patients in three groups (ICM, PCM, and PCP). Using multiple cox regression analysis by controlling for the effect of other factors, our results showed that only patients with ICM were significantly associated with overall survival compared to ICI patients. Interestingly, results from CCA patients in two groups PCM and PCP, changed from significant to non-significant from bivariate analyses compared to multivariable analyses. This may be caused by controlling for other factors in the bivariate analyses, whereas those factors cannot be ignored in multivariable analyses and models which previously found relationships with CCA [25, 28, 29]. Result from our study show that the strongest association was in the ICM group in which patients with a combination of ICCA and MF are most likely to die with 45% mortality (adjusted HR: 1.45; 95% CI: 1.01–2.09) compared to those who had a combination of ICCA and ID. However, when classified by only anatomical characteristics, we found that patients in the ICCA group were most likely to die 49% (HR: 1.49; 95% CI: 1.10–2.04), compared to the DCCA group. Furthermore, this effect was more than for those CCA patients with a combination of anatomical and morphological classifications.

Limitations of the study were the demographic and some health data were self-reported leading to potential bias in the measurement of liver fluke infection, and praziquantel treatment. Self-reporting could lead to underestimates of the size of effects due to an unwillingness to disclose sensitive or personal information. Studies involving survival outcome could be affected by lead time bias. However, our study compared within the same cohort for their survival according to the anatomical and morphological classification. Mean age at diagnosis are similar across the classification group. Therefore, such bias plays no roles in our findings. We also concern about the effect of misclassification on our study due to the limitation in the diagnosis processes. However, misclassification bias may not an issue because there is no evidence of systematic misclassification, i.e., it could occurred but mainly just by chance.

## Conclusions

Among CCA patients receiving surgical treatment with only an anatomical classification, the highest 5-year survival rate was found in DCCA patients, whereas the lowest was in PCCA patients. However, when classified by morphological characteristics, the survival rate was higher than when classified by anatomical features alone especially in patients with a combination of PCCA and ID. The patients in the PCI group had the longest median survival time, whereas the shortest was for patients in the ICM group. When we examined the association between a combination of anatomical and morphological classifications and survival in a high-risk population for CCA, our results revealed that patients with ICM had the strongest association compared to other groups. Based on our results, the treatment plan for CCA patients must be conducted urgently and carefully, especially in patients with PCI who are most likely to have a high survival rate. Screening for CCA is necessary and should be conducted extensively in high risk areas, including all areas throughout Thailand and mainland SE Asia in order to detect people in early stages of the disease thus assisting in prolonging their survival.

## Supplementary Information

**Additional file 1: Table S1**. Incidence rate and median survival time of cholangiocarcinoma patients according to anatomical classification, combination of anatomical and morphological classification separated by stage of cholangiocarcinoma. **Figure S1**. Kaplan-Meier survival estimates of cholangiocarcinoma for anatomical classification separated by stage of cholangiocarcinoma. A Intrahepatic cholangiocarcinoma. B Perihilar cholangiocarcinoma. C Distal cholangiocarcinoma. CCA: Cholangiocarcinoma; ICCA: Intrahepatic CCA; PCCA: Perihilar CCA; DCCA: Distal CCA. **Figure S2**. Kaplan-Meier survival estimates of intrahepatic cholangiocarcinoma classify by morphologically for early and late stage. A Mass-forming. B Periductal-infiltrating. C Intraductal. D Mix morphological. CCA: Cholangiocarcinoma; ICM: Intrahepatic+Mass-forming; ICP: Intrahepatic+Periductal-infiltrating; ICI: Intrahepatic+Intraductal; ICMIX: Intrahepatic+Mix morphological. **Figure S3**. Kaplan-Meier survival estimates of perihilar cholangiocarcinoma classify by morphologically for early and late stage. A Mass-forming. B Periductal-infiltrating. C Intraductal. D Mix morphological. CCA: Cholangiocarcinoma; PCM: Perihilar+Mass-forming; PCP: Perihilar+Periductal-infiltrating; PCI: Perihilar+Intraductal; PCMIX: Perihilar+Mix morphological. **Figure S4**. Kaplan-Meier survival estimates of distal cholangiocarcinoma classify by morphologically for early and late stage. A Mass-forming. B Periductal-infiltrating. C Intraductal. CCA: Cholangiocarcinoma; DCM: Distal+Mass-forming; DCP: Distal+Periductal-infiltrating; DCI: Distal+Intraductal. **Figure S5**. One-year, three-year, and five-year survival rate of CCA according to anatomical classification separated by stage of CCA. CCA: Cholangiocarcinoma; CI: Confidence interval; ICCA: Intrahepatic CCA; PCCA: Perihilar CCA; DCCA: Distal CCA. **Figure S6**. One-year, three-year, and five-year survival rate according to anatomical and morphological classifications for early stage of cholangiocarcinoma patients. CCA: Cholangiocarcinoma; CI: Confidence interval; ICM: Intrahepatic+Mass-forming; ICP: Intrahepatic+Periductal-infiltrating; ICI: Intrahepatic+Intraductal; ICMIX: Intrahepatic+Mix morphological; PCM: Perihilar+Mass-forming; PCP: Perihilar+Periductal-infiltrating; PCI: Perihilar+Intraductal; PCMIX: Perihilar+Mix morphological; DCM: Distal+Mass-forming; DCP: Distal+Periductal-infiltrating; DCI: Distal+Intraductal. **Figure S7**. One-year, three-year, and five-year survival rate according to anatomical and morphological classifications for late stage of cholangiocarcinoma patients. CCA: Cholangiocarcinoma; CI: Confidence interval; ICM: Intrahepatic+Mass-forming; ICP: Intrahepatic+Periductal-infiltrating; ICI: Intrahepatic+Intraductal; ICMIX: Intrahepatic+Mix morphological; PCM: Perihilar+Mass-forming; PCP: Perihilar+Periductal-infiltrating; PCI: Perihilar+Intraductal; PCMIX: Perihilar+Mix morphological; DCM: Distal+Mass-forming; DCP: Distal+Periductal-infiltrating; DCI: Distal+Intraductal.

## Data Availability

All data analyzed during the current study are included in this published article [and its supplementary information file].

## Abbreviations

CCA: Cholangiocarcinoma
US: Ultrasonography
CASCAP: Cholangiocarcinoma Screening and Care Program
ICCA: Intrahepatic CCA
PCCA: Perihilar CCA
DCCA: Distal CCA
MF: Mass-forming
PI: Periductal-infiltrating
ID: Intraductal
ICM: ICCA+MF
ICP: ICCA+PI
ICI: ICCA+ID
ICMIX: ICCA+Mix morphology
PCM: PCCA+MF
PCP: PCCA+PI
PCI: PCCA+ID
PCMIX: PCCA+Mix morphology
DCM: DCCA+MF
DCP: DCCA+PI
DCI: DCCA+ID
CI: Confidence interval
HR: Hazard ratio
