# Supplementary material for: Survival after surgery among patients with cholangiocarcinoma in Northeast Thailand according to anatomical and morphological classification
Source: BMC Cancer. 2021 May 3;21:497. doi: 10.1186/s12885-021-08247-z (PMC8094526; doi:10.1186/s12885-021-08247-z)
Supplement: Supplementary file 1 — Additional file 1: Table S1. Incidence rate and median survival time of cholangiocarcinoma patients according to anatomical classification, combination of anatomical and morphological classification separated by stage of cholangiocarcinoma. Figure S1. Kaplan-Meier survival estimates of cholangiocarcinoma for anatomical classification separated by stage of cholangiocarcinoma. A Intrahepatic cholangiocarcinoma. B Perihilar cholangiocarcinoma. C Distal cholangiocarcinoma. CCA: Cholangiocarcinoma; ICCA: Intrahepatic CCA; PCCA: Perihilar CCA; DCCA: Distal CCA. Figure S2. Kaplan-Meier survival estimates of intrahepatic cholangiocarcinoma classify by morphologically for early and late stage. A Mass-forming. B Periductal-infiltrating. C Intraductal. D Mix morphological. CCA: Cholangiocarcinoma; ICM: Intrahepatic+Mass-forming; ICP: Intrahepatic+Periductal-infiltrating; ICI: Intrahepatic+Intraductal; ICMIX: Intrahepatic+Mix morphological. Figure S3. Kaplan-Meier survival estimates of perihilar cholangiocarcinoma classify by morphologically for early and late stage. A Mass-forming. B Periductal-infiltrating. C Intraductal. D Mix morphological. CCA: Cholangiocarcinoma; PCM: Perihilar+Mass-forming; PCP: Perihilar+Periductal-infiltrating; PCI: Perihilar+Intraductal; PCMIX: Perihilar+Mix morphological. Figure S4. Kaplan-Meier survival estimates of distal cholangiocarcinoma classify by morphologically for early and late stage. A Mass-forming. B Periductal-infiltrating. C Intraductal. CCA: Cholangiocarcinoma; DCM: Distal+Mass-forming; DCP: Distal+Periductal-infiltrating; DCI: Distal+Intraductal. Figure S5. One-year, three-year, and five-year survival rate of CCA according to anatomical classification separated by stage of CCA. CCA: Cholangiocarcinoma; CI: Confidence interval; ICCA: Intrahepatic CCA; PCCA: Perihilar CCA; DCCA: Distal CCA. Figure S6. One-year, three-year, and five-year survival rate according to anatomical and morphological classifications for early stage of cholangio [file 12885_2021_8247_MOESM1_ESM.docx]

**Survival after surgery among patients with cholangiocarcinoma in northeast Thailand according to anatomical and morphological classification**

**Authors:** Chaiwat Tawarungruang^1,2,7^, Narong Khuntikeo^2,3,4^, Nittaya Chamadol^2,3,5^, Vallop Laopaiboon^5^, Jaruwan Thuanman^2,7^, Kavin Thinkhamrop^3,8^, Matthew Kelly^9^, Bandit Thinkhamrop^2,6,7*^

**Affiliations:**

^1^Epidemiology and Biostatistics Program, Faculty of Public Health, Khon Kaen University, Khon Kaen, Thailand.

^2^Cholangiocarcinoma Screening and Care Program (CASCAP), Faculty of Medicine, Khon Kaen University, Khon Kaen, Thailand.

^3^Cholangiocarcinoma Research Institute (CARI), Khon Kaen, Thailand.

^4^Department of Surgery, Faculty of Medicine, Khon Kaen University, Thailand.

^5^Department of Radiology, Faculty of Medicine, Khon Kaen University, Thailand.

^6^Epidemiology and Biostatistics Section, Faculty of Public Health, Khon Kaen University, Thailand.

^7^Data Management and Statistical Analysis Center (DAMASAC), Faculty of Public Health, Khon Kaen University, Thailand.

^8^Health and Epidemiology Geoinformatics Research (HEGER), Faculty of Public Health, Khon Kaen University, Thailand.

^9^Department of Global Health, Research School of Population Health, Australian National University, Canberra, Australia.

Correspondence and requests for materials should be addressed to B.T. (e-mail: bandit@kku.ac.th)

**Supplementary Table**

Table S1. Incidence rate and median survival time of cholangiocarcinoma patients according to anatomical classification, combination of anatomical and morphological classification separated by stage of cholangiocarcinoma

| **Stage of CCA** | **Number** | **Person-months** | **IR per 100** | **Median time** | **95% CI** |
| --- | --- | --- | --- | --- | --- |
| Early stage |  |  |  |  |  |
| Overall | 259 | 8,118 | 1.4 | 60.1 | 49.5-70.8 |
| Anatomical classification |  |  |  |  |  |
| ICCA | 150 | 4,789 | 1.3 | 63.4 | 51.4-75.4 |
| PCCA | 82 | 2,560 | 1.3 | 53.3 | 38.0-68.6 |
| DCCA | 27 | 769 | 2.0 | 32.0 | 12.5-51.4 |
| Combination of anatomical and morphological classification |  |  |  |  |  |
| ICM | 80 | 2,438 | 1.6 | 52.6 | 35.4-69.9 |
| ICP | 23 | 755 | 1.1 | - | - |
| ICI | 44 | 1,497 | 1.0 | 63.4 | 48.4-70.1 |
| ICMIX | 3 | 98 | 0.0 | - | - |
| PCM | 5 | 123 | 2.4 | 42.6 | 28.5-56.6 |
| PCP | 44 | 1,351 | 1.5 | 44.1 | 30.2-58.0 |
| PCI | 30 | 1,008 | 1.1 | 60.1 | 55.4-64.9 |
| PCMIX | 3 | 78 | 0.0 | - | - |
| DCM | 8 | 114 | 4.4 | 5.4 | 11.2-22.0 |
| DCP | 11 | 416 | 1.2 | - | - |
| DCI | 8 | 238 | 2.1 | 26.7 | 00.2-53.2 |
| Late stage |  |  |  |  |  |
| Overall | 487 | 7,526 | 5.4 | 11.4 | 09.8-12.9 |
| Anatomical classification |  |  |  |  |  |
| ICCA | 222 | 2,925 | 6.5 | 8.4 | 06.2-10.6 |
| PCCA | 212 | 3,505 | 5.1 | 14.1 | 11.0-17.2 |
| DCCA | 53 | 1,096 | 3.2 | 20.6 | 11.6-29.5 |
| Combination of anatomical and morphological classification |  |  |  |  |  |
| ICM | 158 | 1,935 | 7.3 | 8.1 | 05.7-10.4 |
| ICP | 24 | 406 | 4.7 | 12.2 | 03.5-27.9 |
| ICI | 26 | 388 | 5.4 | 8.9 | 00.6-18.5 |
| ICMIX | 14 | 196 | 4.6 | 7.5 | 03.9-11.0 |
| PCM | 53 | 867 | 5.1 | 14.8 | 07.0-22.5 |
| PCP | 109 | 1,722 | 5.7 | 10.7 | 05.8-15.6 |
| PCI | 21 | 389 | 3.9 | 13.9 | 06.2-21.7 |
| PCMIX | 29 | 527 | 4.0 | 16.9 | 06.5-27.3 |
| DCM | 24 | 561 | 2.7 | 23.4 | 19.6-27.2 |
| DCP | 23 | 404 | 4.0 | 12.4 | 06.5-18.2 |
| DCI | 6 | 131 | 3.0 | 11.3 | 03.6-19.1 |

CCA: Cholangiocarcinoma; ICCA: Intrahepatic CCA; PCCA: Perihilar CCA; DCCA: Distal CCA; ICM: ICCA+Mass-forming; ICP: ICCA+Periductal-infiltrating; ICI: ICCA+Intraductal; ICMIX: ICCA+Mix morphological; PCM: PCCA+Mass-forming; PCP: PCCA+Periductal-infiltrating; PCI: PCCA+Intraductal; PCMIX: PCCA+Mix morphological; DCM: DCCA+Mass-forming; DCP: DCCA+Periductal-infiltrating; DCI: DCCA+Intraductal; IR: Incidence rate; CI: Confidence interval.

**Supplementary Figure**

**C**

**B**

**A**

Figure S1. Kaplan-Meier survival estimates of cholangiocarcinoma for anatomical classification separated by stage of cholangiocarcinoma. **A** Intrahepatic cholangiocarcinoma. **B** Perihilar cholangiocarcinoma. **C** Distal cholangiocarcinoma. CCA: Cholangiocarcinoma; ICCA: Intrahepatic CCA; PCCA: Perihilar CCA; DCCA: Distal CCA.

**D**

**C**

**B**

**A**

Figure S2. Kaplan-Meier survival estimates of intrahepatic cholangiocarcinoma classify by morphologically for early and late stage. **A** Mass-forming. **B** Periductal-infiltrating. **C** Intraductal. **D** Mix morphological. CCA: Cholangiocarcinoma; ICM: Intrahepatic+Mass-forming; ICP: Intrahepatic+Periductal-infiltrating; ICI: Intrahepatic+Intraductal; ICMIX: Intrahepatic+Mix morphological.

**D**

**C**

**B**

**A**

Figure S3. Kaplan-Meier survival estimates of perihilar cholangiocarcinoma classify by morphologically for early and late stage. **A** Mass-forming. **B** Periductal-infiltrating. **C** Intraductal. **D** Mix morphological. CCA: Cholangiocarcinoma; PCM: Perihilar+Mass-forming; PCP: Perihilar+Periductal-infiltrating; PCI: Perihilar+Intraductal; PCMIX: Perihilar+Mix morphological.

**C**

**B**

**A**

Figure S4. Kaplan-Meier survival estimates of distal cholangiocarcinoma classify by morphologically for early and late stage. **A** Mass-forming. **B** Periductal-infiltrating. **C** Intraductal. CCA: Cholangiocarcinoma; DCM: Distal+Mass-forming; DCP: Distal+Periductal-infiltrating; DCI: Distal+Intraductal.

| **Anatomical classification** | **Survival rate** | | |  | **95% CI** | | |  |
| --- | --- | --- | --- | --- | --- | --- | --- | --- |
| **Early stage** |  | | |  |  |  |  |  |
| 1-year |  | | |  |  |  |  |  |
| Overall | 82.2 | | |  |  |  | 77.0-86.4 |  |
| ICCA | 84.0 | | |  |  |  | 77.1-89.0 |  |
| PCCA | 82.9 | | |  |  |  | 72.9-89.5 |  |
| DCCA | 70.4 | | |  |  |  | 49.4-83.9 |  |
| 3-year |  | | |  |  |  |  |  |
| Overall | 60.6 | | |  |  |  | 54.1-66.6 |  |
| ICCA | 61.3 | | |  |  |  | 52.6-68.9 |  |
| PCCA | 64.1 | | |  |  |  | 51.8-74.0 |  |
| DCCA | 46.4 | | |  |  |  | 26.6-64.1 |  |
| 5-year |  | | |  |  |  |  |  |
| Overall | 50.6 | | |  |  |  | 42.8-58.0 |  |
| ICCA | 54.2 | | |  |  |  | 43.9-63.4 |  |
| PCCA | 46.7 | | |  |  |  | 31.5-60.5 |  |
| DCCA | 39.8 | | |  |  |  | 19.9-59.1 |  |
|  |  | | |  |  |  |  |  |
|  |  | |  |  |  |  |  |  |
|  |  | 0 30 60 90 | | | | | | |
| **Late stage** |  | | |  |  |  |  |  |
| 1-year |  | | |  |  |  |  |  |
| Overall | 48.7 | | |  |  |  | 44.2-53.0 |  |
| ICCA | 40.1 | | |  |  |  | 33.6-46.5 |  |
| PCCA | 54.3 | | |  |  |  | 47.3-60.7 |  |
| DCCA | 62.3 | | |  |  |  | 47.8-73.8 |  |
| 3-year |  | | |  |  |  |  |  |
| Overall | 14.7 | | |  |  |  | 11.4-18.4 |  |
| ICCA | 12.2 | | |  |  |  | 07.9-17.5 |  |
| PCCA | 12.3 | | |  |  |  | 07.7-18.1 |  |
| DCCA | 32.7 | | |  |  |  | 20.4-45.6 |  |
| 5-year |  | | |  |  |  |  |  |
| Overall | 9.5 | | |  |  |  | 05.6-14.6 |  |
| ICCA | 4.6 | | |  |  |  | 00.7-14.8 |  |
| PCCA | 8.2 | | |  |  |  | 03.9-14.4 |  |
| DCCA | 32.7 | | |  |  |  | 20.4-45.6 |  |
|  |  | | |  |  |  |  |  |
|  |  | |  |  |  |  |  |  |
|  |  | 0 30 60 90 | | | | | | |
|  |  |  | | Survival rate | | |  |  |

Figure S5. One-year, three-year, and five-year survival rate of CCA according to anatomical classification separated by stage of CCA. CCA: Cholangiocarcinoma; CI: Confidence interval; ICCA: Intrahepatic CCA; PCCA: Perihilar CCA; DCCA: Distal CCA.

| **Combination of anatomical and morphological classification** | **Survival rate** | | |  | **95% CI** | | |
| --- | --- | --- | --- | --- | --- | --- | --- |
| **Early stage** |  | | |  |  |  |  |
| 1-year |  | | |  |  |  |  |
| Overall | 82.2 | | |  |  |  | 77.0-86.4 |
| ICM | 77.5 | | |  |  |  | 66.7-85.2 |
| ICP | 91.3 | | |  |  |  | 69.5-97.8 |
| ICI | 90.9 | | |  |  |  | 77.6-96.5 |
| ICMIX | 100 | | |  |  |  | - |
| PCM | 80.0 | | |  |  |  | 20.4-96.9 |
| PCP | 77.3 | | |  |  |  | 61.9-87.1 |
| PCI | 90.0 | | |  |  |  | 72.1-96.7 |
| PCMIX | - | | |  |  |  | - |
| DCM | 50.0 | | |  |  |  | 15.2-77.5 |
| DCP | 81.8 | | |  |  |  | 44.7-95.1 |
| DCI | 75.0 | | |  |  |  | 31.5-93.1 |
| 3-year |  | | |  |  |  |  |
| Overall | 60.6 | | |  |  |  | 54.1-66.6 |
| ICM | 56.2 | | |  |  |  | 44.3-66.4 |
| ICP | 62.3 | | |  |  |  | 38.1-79.3 |
| ICI | 67.8 | | |  |  |  | 50.7-80.0 |
| ICMIX | - | | |  |  |  | - |
| PCM | 53.3 | | |  |  |  | 06.8-86.3 |
| PCP | 57.8 | | |  |  |  | 40.5-71.7 |
| PCI | 71.7 | | |  |  |  | 51.0-84.8 |
| PCMIX | - | | |  |  |  | - |
| DCM | 37.5 | | |  |  |  | 08.7-67.4 |
| DCP | 63.6 | | |  |  |  | 29.7-84.5 |
| DCI | 37.5 | | |  |  |  | 08.7-67.4 |
| 5-year |  | | |  |  |  |  |
| Overall | 50.6 | | |  |  |  | 42.8-58.0 |
| ICM | 47.3 | | |  |  |  | 33.5-59.8 |
| ICP | 62.3 | | |  |  |  | 38.1-79.3 |
| ICI | 60.2 | | |  |  |  | 38.8-76.2 |
| ICMIX | - | | |  |  |  | - |
| PCM | 53.3 | | |  |  |  | 06.8-86.3 |
| PCP | 42.2 | | |  |  |  | 22.7-60.4 |
| PCI | 56.3 | | |  |  |  | 30.4-75.8 |
| PCMIX | - | | |  |  |  | - |
| DCM | 37.5 | | |  |  |  | 08.7-67.4 |
| DCP | 50.9 | | |  |  |  | 18.2-76.6 |
| DCI | 37.5 | | |  |  |  | 08.7-67.4 |
|  |  | | |  |  |  |  |
|  |  | |  |  |  |  |  |
|  |  | 0 30 60 90 | | | | | |
|  |  |  | | Survival rate | | |  |

Figure S6. One-year, three-year, and five-year survival rate according to anatomical and morphological classifications for early stage of cholangiocarcinoma patients. CCA: Cholangiocarcinoma; CI: Confidence interval; ICM: Intrahepatic+Mass-forming; ICP: Intrahepatic+Periductal-infiltrating; ICI: Intrahepatic+Intraductal; ICMIX: Intrahepatic+Mix morphological; PCM: Perihilar+Mass-forming; PCP: Perihilar+Periductal-infiltrating; PCI: Perihilar+Intraductal; PCMIX: Perihilar+Mix morphological; DCM: Distal+Mass-forming; DCP: Distal+Periductal-infiltrating; DCI: Distal+Intraductal.

| **Combination of anatomical and morphological classification** | **Survival rate** | | |  | **95% CI** | | |
| --- | --- | --- | --- | --- | --- | --- | --- |
| **Late stage** |  | | |  |  |  |  |
| 1-year |  | | |  |  |  |  |
| Overall | 48.7 | | |  |  |  | 44.2-53.0 |
| ICM | 36.7 | | |  |  |  | 29.3-44.2 |
| ICP | 54.2 | | |  |  |  | 32.7-71.4 |
| ICI | 46.2 | | |  |  |  | 26.6-63.6 |
| ICMIX | 42.9 | | |  |  |  | 17.7-66.0 |
| PCM | 56.6 | | |  |  |  | 42.3-68.7 |
| PCP | 45.9 | | |  |  |  | 36.3-55.0 |
| PCI | 61.9 | | |  |  |  | 38.1-78.8 |
| PCMIX | 75.9 | | |  |  |  | 55.9-87.7 |
| DCM | 75.0 | | |  |  |  | 52.6-87.9 |
| DCP | 52.2 | | |  |  |  | 30.5-70.0 |
| DCI | 50.0 | | |  |  |  | 11.1-80.4 |
| 3-year |  | | |  |  |  |  |
| Overall | 14.7 | | |  |  |  | 11.4-18.4 |
| ICM | 8.7 | | |  |  |  | 04.6-14.3 |
| ICP | 18.5 | | |  |  |  | 05.9-36.5 |
| ICI | 26.4 | | |  |  |  | 11.4-44.1 |
| ICMIX | 42.9 | | |  |  |  | 17.7-66.0 |
| PCM | 14.3 | | |  |  |  | 05.6-27.0 |
| PCP | 08.8 | | |  |  |  | 04.1-15.9 |
| PCI | 24.7 | | |  |  |  | 08.4-45.5 |
| PCMIX | 75.9 | | |  |  |  | 55.9-87.7 |
| DCM | 35.4 | | |  |  |  | 17.1-54.4 |
| DCP | 29.8 | | |  |  |  | 12.9-48.9 |
| DCI | 33.3 | | |  |  |  | 04.6-67.6 |
| 5-year |  | | |  |  |  |  |
| Overall | 9.5 | | |  |  |  | 05.6-14.6 |
| ICM | 3.7 | | |  |  |  | 00.5-12.6 |
| ICP | 18.5 | | |  |  |  | 05.9-36.5 |
| ICI | 26.4 | | |  |  |  | 11.4-44.1 |
| ICMIX | 42.9 | | |  |  |  | 17.7-66.0 |
| PCM | 14.3 | | |  |  |  | 05.6-27.0 |
| PCP | 5.9 | | |  |  |  | 02.1-12.5 |
| PCI | 24.7 | | |  |  |  | 08.4-45.5 |
| PCMIX | 75.9 | | |  |  |  | 55.9-87.7 |
| DCM | 35.4 | | |  |  |  | 17.1-54.4 |
| DCP | 29.8 | | |  |  |  | 12.9-48.9 |
| DCI | 33.3 | | |  |  |  | 04.6-67.6 |
|  |  | | |  |  |  |  |
|  |  | |  |  |  |  |  |
|  |  | 0 25 50 75 | | | | | |
|  |  |  | | Survival rate | | |  |

Figure S7. One-year, three-year, and five-year survival rate according to anatomical and morphological classifications for late stage of cholangiocarcinoma patients. CCA: Cholangiocarcinoma; CI: Confidence interval; ICM: Intrahepatic+Mass-forming; ICP: Intrahepatic+Periductal-infiltrating; ICI: Intrahepatic+Intraductal; ICMIX: Intrahepatic+Mix morphological; PCM: Perihilar+Mass-forming; PCP: Perihilar+Periductal-infiltrating; PCI: Perihilar+Intraductal; PCMIX: Perihilar+Mix morphological; DCM: Distal+Mass-forming; DCP: Distal+Periductal-infiltrating; DCI: Distal+Intraductal.
